# Histone Signatures Predict Therapeutic Efficacy in Breast Cancer

**DOI:** 10.1109/OJEMB.2020.2967105

**Published:** 2020-01-17

**Authors:** Shamim A. Mollah, Shankar Subramaniam

**Affiliations:** 1 ^1^Bioinformatics & Systems Biology ProgramThe University of California San Diego 8784 La JollaCA92093USA; 2 ^2^Department of GeneticsWashington University 553822 St. LouisMO63130USA; 3 ^3^Departments of Bioengineering, Cellular & Molecular Medicine and Computer Science & EngineeringThe University of California San Diego 8784 La JollaCA92093USA

**Keywords:** BRD4, breast cancer, chromatin remodeling, flavopiridol, histone modification

## Abstract

*Objective:* Regulatory abnormalities caused by chromatin modifications are being increasingly recognized as contributors to cancer. While many molecularly targeted drugs have the potential to revert these modifications, their precise mechanism of action in cellular reprogramming is not known. *Methods:* To address this, we introduce an integrated phosphoprotein-histone-drug network (iPhDNet) approach to generate “global chromatin fingerprints of histone signatures.” The method integrates proteomic/phosphoproteomic, transcriptomic and regulatory genomic data to provide a causal mechanistic network and histone signatures of drug response. *Results:* We demonstrate the utility of iPhDNet in identifying H3K27me3K36me3 histone mark as a key fingerprint of response, mediated by chromatin remodelers BRD4, NSD3, EZH2, and a proto-oncogene MYC when treated with CDK inhibitors. *Conclusions:* We construct a regulatory network of breast cancer response to treatment and show that histone H3K27me3K36me3 status changes, driven by the BRD4/MYC pathway, upon treatment with drugs are hallmarks of response to treatment.

## I. Introduction

Cancer arises due to aberrant genetic [1] and epigenetic dysregulation [2], [3], causing normal cells to proliferate. There is increasing evidence that several regulatory abnormalities are caused through post-translational modification (PTM) of histones [4] resulting from a variety of covalent modifications including, phosphorylation, methylation, acetylation, and ubiquitination at the N-terminal tails of histones. A single or combinatorial set of these modifications on one or more histone tail comprises a ‘histone code’ [5] which greatly influences the control of the chromatin structure, function, and interactions leading to altered downstream cellular processes. The histone codes play key roles in the regulation of gene expression, switching genes on and off by making the DNA accessible/inaccessible to the transcriptional machinery. In contrast to the irreversible genomic mutations that activate oncogenes or inactivate tumor suppressor genes in cancer, histone modifications are reversible and can be used as potential biomarkers for normal or cancer state of cells, and as markers of drug response. Furthermore, histone modification enzymes themselves can be targets of therapy if their specific roles are understood [6], [7].

In a recent large-scale initiative, Library of Integrated Network-Based Cellular Signatures (LINCS) (http://www.lincsproject.org), has carried out multi-omics characterization of response of five cancer cells to 31 drugs, through measurement of phosphoproteins (P100) [8], transcripts (L1000) [9], and global chromatin profiles (GCP) [10]. Some of these measurements were carried out at multiple time points post-treatment of cells.  The P100 and GCP are Mass Spectrometry (MS)-based targeted proteomics assays that include a representative set of phosphopeptides, and different combinations of histone modifications treated by multiple drugs respectively. L1000 data was generated using landmark transcript probes [9] of genes which were invariant across cell states. This multi-omics study offers the scope for obtaining key signatures of drug response based on detailed mechanisms reconstructed from the measurements. Specifically, we present a novel integrated approach for identifying cellular fingerprints of response to drug treatment in breast cancer cells.  Our methods identify histone modification fingerprints, which are endpoints of complex signaling events following drug treatment. The epigenetic changes and the concomitant chromatin topology changes, caused by these histone signatures, reflect the altered cellular state.

For characterization of the epigenetic fingerprint responses, we used the MCF7 cell line from the LINCS study that profiled 96 phosphopeptides at three time points and 60 histone marks profiled 24 hours after treatment with 31 established drugs. This type of high-dimensional data represents significant challenges for analyzing the pattern of drug responses affecting GCP, and deciphering pathways that are causally involved in responses leading to specific GCP. We approached this from the perspective of data and dimension reduction in order to develop mechanistic models of drug response through GCP fingerprints. In the following sections, we describe the integrated network we developed for analyzing the LINCS breast cancer data to 1) uncover the number of distinct ways in which drugs relate to GCP; 2) decipher the unique phosphoproteins networks and pathways that describe histone response to 31 drug treatments; and 3) identify mechanisms involving phosphoproteins regulating a wide range of cellular processes (growth, proliferation and cell division) and gene activity states. Our results demonstrate fingerprints of GCP that comprehensively describe the drug response in cancer cells and further help elucidate the detailed causal mechanisms that lead to these epigenetic profiles.

## II. Results

### A. Four Pathway-Based Histone Signatures Constitute “Histone Signatures” Responding to Drug Treatment

**Fig. 1.**
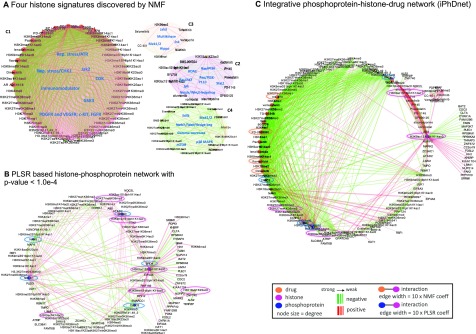
A 3-dimensional view of molecular interactions of phosphoproteins-histones-drugs generated by integrating the histone signatures with the histone-protein interaction network. (**A**) Four “histone signatures” are obtained by NMF clustering of GCP at 24-hour post-treatment. Drugs and histones are depicted by orange and magenta nodes respectively, the color of edges signifies whether the interaction between a drug and a histone resulted in elevated (red) or reduced (green) histone level. (**B**) Histone-phosphoprotein interaction network using a PLSR prediction model. Histones and phosphoproteins are depicted by magenta and blue nodes respectively, the color of edges depicts whether the interaction between a phosphoprotein and a histone is positively (red) or negatively (green) correlated. (**C**) Integrated phosphoproteins-histones-drug network (iPhDNet). iPhDNet shows enrichment of modification levels on H3K9ac1S10ph1K14ac0, H3K56me2, H3K27me3K36me3, H3K18ac0K23ub1 histone codes, acting as highly connected nodes (hubs) and positively induced by various drugs affecting enriched phosphoproteins including BRD4, ATAD2, and NOLC1. The strength of an interaction is captured by the width of an edge.

In order to identify histone signatures that are elicited by the drugs through canonical signaling pathways, we investigated the relationships between the 31 drugs targeting serine-threonine kinases in the breast cancer line (MCF7), using the resulting GCP response at 24 hours (**Supplementary Table 1**). We first calculated the histone code fold changes by accounting for their differential modifications i.e., changes in histone levels from pre-treatment (MCF7 treated with DMSO) to post-treatment (MCF7 treated with a specific drug) state. Using a non-negative matrix factorization (NMF) method on these histone code fold changes, we identified four pathway-based functional histone modules c1, c2, c3 and c4 (Fig. 1A) and refer to them as “histone signatures” that characterize the response to drugs (**Supplementary Fig. S1D**).

To provide a comprehensive mapping of these histone signatures to drugs with respect to their shared signaling pathways, we generated a molecular network consisting of 91 nodes (comprising histone codes and drugs) and 554 edges (node interactions). Coefficients generated from the assignments of each histone signature profile to the drug prototypes are used to represent the strength/weight of the interactions between a histone code and a drug (**Supplementary Fig. S1A, B**). We observed all cyclin-dependent kinase (CDK) inhibitors and replication stress inhibitors were grouped with the same c1 histone signature. We observed similar groupings for the c2, c3 and c4 signatures associating specific histone codes with specific drugs (Fig. 1A). **Supplementary Table 2** summarizes the grouping of these drugs and histones into their respective signatures.

### B. A Quantitative Model for Enriched Phosphoproteins Contributing to Histone Codes

Next, we sought to identify the phosphoprotein networks representing various interactions among the enriched phosphoproteins and histone codes. Using principal least squares regression (PLSR) on P100 phosphoproteins and GCP responses at 24 hours after treatment with the 31 drugs (**Supplementary Table 1**), we generated a systemic model where each histone code is considered as an outcome/response to combined influences (i.e., coefficients) of all phosphoproteins. Each coefficient represents the contribution of individual phosphoprotein toward the level of a histone code. Using a t-test hypothesis testing with a p-value <1.0e-4 (see Methods), our model generated a reduced histone-phosphoprotein network comprised of 113 nodes, representing histone codes and phosphoproteins and 230 edges (interactions between them) (Fig. 1B). The model performance is depicted in **Supplementary Fig S2.** Our results showed H3K27me3K36me3, H3K9ac1S10ph1K14ac0, H3K56me2, and H3K18ac0K23ub1 as highly connected histone codes (hubs with the highest degree), influenced by the statistically enriched phosphoproteins: BRD4, ATAD2, NOLC1, SRRM2, and CASC3. **Supplementary Table 3** summarizes the characteristics of these enriched phosphoproteins.

### C. A 3-Dimensional View of Molecular Interactions Among Drugs-Phosphoproteins-Histones

To further elucidate the influence of specific drugs on phosphoproteins and downstream histone codes, we developed a 3D view of the molecular interactions (phosphoproteins-drugs-histones) by integrating histone signatures with the drug-phosphoprotein interaction network resulting in an integrated phosphoproteins-histones-drugs network (iPhDNet). This network consists of 144 nodes and 742 interactions (Fig. 1C).

Using the iPhDNet as a quantitative atlas of global chromatin profile fingerprints, we then generated hypotheses linking drugs, pathways, phosphoproteins, and histones, to understand drug response pathways in breast cancer. These chromatin profile fingerprints revealed an overall reduction in histone levels in active marks such as methylation of H3K36, H3K4, and acetylation of H3K9 when treated with these drugs, consistent with previous studies where these marks were elevated in various untreated cancer cell lines [4], [14], [15]. We observed that reduction of phosphoprotein level in SRRM2 was positively correlated (p-val <2.7e-04) with H3K4me1 and H3K4me3 when treated with drugs that belonged to c1 signature histone module (Supplementary Table 2). While H3K27me3 is a repressive histone mark associated with transcriptionally silenced chromatin in most cancers [4], [14], [15], analysis from iPhDNet revealed inhibitory effects of drugs on H3K27me3 in breast cancer, consistent with the prior studies [16], [17]. Likewise, ABI1, an adaptor protein involved in cell migration, along with its downstream effector phospho-Akt (p-Akt), has been implicated in the spread of breast cancer [18]; is positively correlated with reduced H3K27me3K36me3 (p-val <7.02e-05) when inhibited by CDK inhibitor flavopiridol.

Additionally, we observed significant associations (p-val <7.5e-05) of ABI1, BRD4, NOLC1, ATAD2, and SRRM2 with H3K27me3K36me3 when treated with CDK inhibitors flavopiridol, dinaciclib, and PD-033291 (Fig. 1C). Collectively, iPhDNet shows that inhibiting ABI1, BRD4, NOLC1, ATAD2, and SRRM2 with the help of CDK inhibitors may be sufficient to induce the heterochromatin state when the repressive mark H3K27me3 colocalizes with the active mark H3K36me3. This finding suggests that for a stable reversion of epigenetic silencing state in breast cancer, H3K36me3 may dictate a reversal from the malignant euchromatin to normal heterochromatin.

### D. FLAVOPIRIDOL AND DINACICLIB EMERGE AS POTENTIAL CDK MEDIATED THERAPEUTICS IN BREAST CANCER

**Fig. 2.**
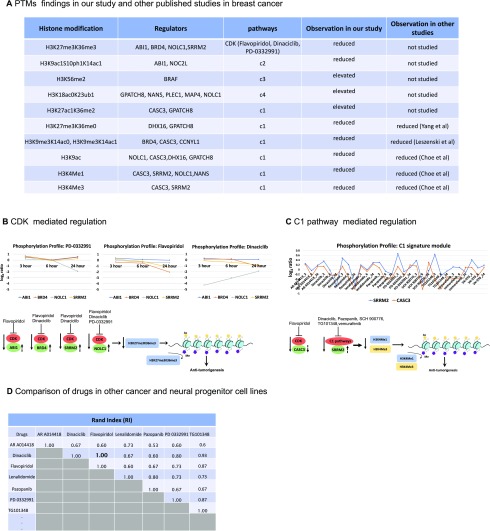
Flavopiridol and dinaciclib emerge as potential CDK mediated therapeutics in breast cancer. (**A**) Summary of PTM results showing consistency of our findings with other reports that interrogated specific PTMs in breast cancers. (**B**) CDK mediated regulation in flavopiridol, dinaciclib, and PD-0332991. Top: phosphorylation profiles depict changes in phosphoprotein values (log2 scale) for ABI1, BRD4, NOLC1 and SRRM2 over 3, 6, 24 hour when treated with these drugs. Bottom: suggested regulation mechanisms for these phosphorylation profiles and their corresponding H3K27me3K36me3 values resulting in anti-tumorigenesis events. Flat bars indicate inhibition of CDK by these drugs causing reduced values (down arrow) for these phosphoproteins and the corresponding H3K27me3K36me3 status. The dashed arrows indicate these phosphoproteins are positively (up arrow) correlated with the H3K27me3K36me3 status. (**C**) Showing multiple phosphosignaling pathways regulated by the specific drugs in C1 “histone signature”. Top: phosphorylation profiles of CASC3 and SRRM over 3, 6, 24 hour when treated with each of these C1 drugs. Bottom: suggested regulation mechanisms show reduction of phosphoprotein values of CASC3 and SRRM2 as well as reduction in H3K4me1 and H3K4me3 resulting in anti-tumorigenesis events. SRRM2 is positively correlated (dashed up arrow), CASC3 is negatively correlated (dashed down arrow) with H3K4me1 and H3K4me3 status. (**D**) Comparison of cluster similarity between paired drugs showing an exact match (1.0) between flavopiridol and dinaciclib.

To examine the validity of the identified enriched phosphoproteins mediated by specific drugs, we compared our findings with prior experiments on the identification of various histone codes in breast cancer. A summary of our PTM findings is provided in a table (Fig. 2a). While the studies above have investigated the modification of H3K27me3 and H3K36me3, combinatorial assembly of repressive H3K27me3, and active H3K36me3 marks (H3K27me3K36me3) has not been studied in cancer. Hence, we further analyzed the molecular mechanisms associated with H3K27me3K36me3 modulation to identify potential targets for therapeutic interventions in breast cancer.

Since H3K27me3K36me3 belonged to C1 histone signature, we considered evaluating the effect of drugs in that signature on the enriched phosphoproteins. Using the phosphorylation status at 3, 6, and 24 hours of these enriched phosphoproteins and their interactions profiles from iPhDNet, we postulated the anti-tumorigenic effects of these inhibitors modulating H3K27me3K36me3 (Fig. 2B), H3K4me1, and H3K4me3 (Fig. 2C). We found that flavopiridol and dinaciclib induced the same regulatory pathways, suggesting similar therapeutic responses.

Flavopiridol is a CDK inhibitor, with high selectivity for CDK9, used in phase II clinical trial for the treatment of relapsed/refractory lymphoma or multiple myeloma [19]. Similarly, dinaciclib is a highly potent CDK inhibitor with selectivity for CDK1, CDK2, CDK5, and CDK9 [20] in phase III clinical trials for the treatment of refractory chronic lymphocytic leukemia. To evaluate the concordance of these two inhibitors, we computed the RI on drug assignments to see how many drugs were grouped in the same histone signatures across six cell lines: breast (MCF7), pancreas (YAPC), skin (A375), lung (A549), prostate (PC3) and neural progenitor cell line (NPC). The analysis assigned flavopiridol and dinaciclib in the same histone modules across all six cell lines with a RI score of 1 (Fig. 2D). A Pearson correlation analysis on the P100 phosphoprotein data further supported the concordance between flavopiridol and dinaciclib, showing a strong correlation between the two drug responses at 3 to 6 hour (r = 0.59) and 6 to 24 hour (r = 0.69) (**Supplementary Fig. S3A, B**). A linear regression analysis of histone expressions at 24 hours showed similar treatment effects between flavopiridol and dinaciclib (**Supplementary Fig. S3C**). Additional support is provided by a comparative structural analysis study [21] that indicates a similar affinity toward acetylated lysine (KAc) binding site of bromodomain (BRD) for flavopiridol and dinaciclib.

### E. Mechanistic Causal Network (MCN) Reconstruction Supports BRD4 Mediated Cell Cycle Arrest Caused by Impaired Transcriptional Elongation

**Fig. 3.**
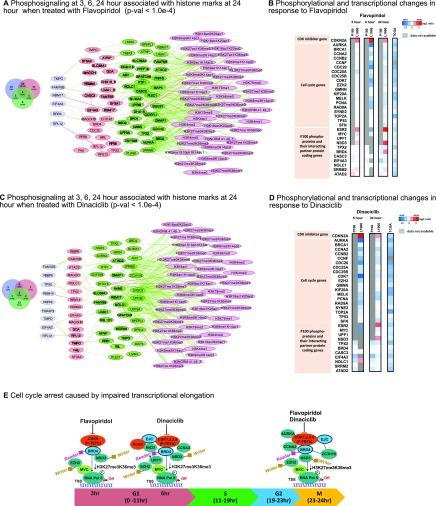
Mechanistic causal network (MCN) reconstruction supports BRD4 mediated cell cycle arrest. (A) MCN reconstruction for enriched phosphoproteins (p-val <1.0e-4) upon flavopiridol treatment. This network is obtained by mapping enriched phosphoproteins with their interaction partners (first level) using STRINGdb protein-protein interactions (PPI) at 3, 6, and 24 hour. The distribution for these enriched phosphoproteins and their interaction partners at each time point is shown in the Venn diagram (left). The colors of phosphoproteins and their partner proteins in MCN correspond to the colors of the Venn diagram at each time point (blue for 3, red for 6, and green 24 hour). (B) Phosphorylation changes of proteins and transcriptional changes of 31 functionally significant genes in response to flavopiridol. Gene expression of CDK inhibitor gene *CDKN2A* is upregulated (red) while gene expressions of all cell cycle genes are downregulated (blue) in L1000 data across all time points 3, 6, 24 hour. Transcriptomic expressions from TCGA data for these genes show down-regulation (blue). The unavailable gene coding phosphoproteins in P100 data are marked gray. (C) A similar mechanistic causal network reconstruction for enriched phosphoproteins after dinaciclib treatment is obtained using the protocol described in A. (D) Similarly, phosphorylation and transcriptional changes of the same phosphoproteins and genes in B, in response to dinaciclib. (E) Demonstrating possible cell cycle arrest mechanisms caused by transcriptional elongation of the participating regulators over various time points corresponds to cell cycle stages: gap phases (G1, G2), and mitotic phase (M). Inhibitory effects of flavopiridol and dinaciclib on BRD4 result in reduced H3K36me3 level caused by synergetic interactions among BRD4, *NSD3*, *EZH2*, *MYC* and EJC complex. Inhibition (blue) of BRD4 (a chromatin reader) impairs the catalytic activity of CDK9 ability to bind to P-TEFb, a subunit of the CDK9. CDK9 acts as a cofactor of *MYC*-dependent stimulation of responsive genes *NSD3* (a chromatin writer), *EZH2* (a chromatin writer), *AURKA*, and *CCNA2*. *MYC* modulates chromatin context surrounding these responsive genes and H3K27me3K36me3 histone marks and influences cell cycle arrest by blocking RNA polymerase elongation process.

To gain mechanistic insights into H3K27me3K36me3 mediated regulation by flavopiridol and dinaciclib, we reconstructed mechanistic causal networks (MCN) that shows the regulatory machinery involving the enriched phosphoproteins measured at varying time points (Fig. 3A**,** C). Our results showed BRD4, TMPO, FAM76B, and RBM17 in flavopiridol and TMPO, FAM76B, and TPX2 in dinaciclib remained enriched across 3, 6, and 24-hour time points. The network showed binding of BRD4/NSD3 consistent with a previous study [22], where reduced H3K36 methylation was a result of the depletion of BRD4 or NSD3. NSD3 is a methyltransferase that binds to BRD4 complexes at the promoter region to regulate levels of H3K36me3, affecting DNA repair, transcription initiation, and elongation/termination processes [23], [24].

To further investigate the mechanisms by which this BRD4/NSD3 complex contributes to mediating cell cycle progression through the recruitment of H3K36me3 and binding to upstream regulators/cofactors, we performed enrichment analyses on the genes representing these phosphoproteins using the Enrichr tool [25]. This identified *MYC*, *POU5F1* (*OCT*4), *ESR2*, *UPF1*, and *BRCA1* as commonly enriched upstream/core regulators of phosphoproteins for flavopiridol and dinaciclib. The analysis showed interactions between spliceosome mediated activities through the core regulators: *E2F4*, *UPF1*, *ILF3*, and *SMARCA4*, and the components of exon junction complex (EJC); interactions among the mitotic regulators (*TPX2*, *AURKA*) with *TP53* activity and *ATAD2* that formed a cluster, regulating cell cycle through alternative splicing. The analysis revealed TPX2, AURKA, and EJC complex as potential substrates of positive transcription elongation factor (P-TEFb), through indirect binding with BRD4.

We next performed transcriptomic analyses using L1000 data (**Supplementary Table 4**) on genes representing the enriched phosphoproteins to capture in vitro gene activity levels. We examined CDK inhibitor genes, and genes coding for parent proteins of enriched phosphoproteins associated with H3K27me3K36me3 mark in the MCF7 cell line. We identified 31 genes (Fig. 3B**,** D). Together, the results from these transcriptomic analyses corroborate our proteomics conclusions. **Supplementary Table 5** summarizes MCN and enrichment analysis results.

Next, we investigated how flavopiridol and dinaciclib lead to preferential loss of BRD4/NSD3, impacting the oncogene *MYC*, thereby, promoting cell cycle arrest in breast cancer. Based on our results and the evidence from previous studies [26], [27], [24], [28], we postulate that cell cycle arrest associated with the reduced H3K27me3K36me3 phenotype occurs through the following mechanism: 1) flavopiridol and dinaciclib inhibit BRD4, 2) as a result, H3K36me3 level is reduced through BRD4's interacting partner NSD3, 3) reduction of BRD4 then impairs the catalytic activity of CDK9's ability to bind to positive transcription elongation factor b (P-TEFb), which is sequestered by 7SK snRNP to acetylated chromatin at the MYC locus, 4) this suppresses P-TEFb's phosphorylation at serine 2 of the Pol II carboxyl-terminal domain (CTD) and DSIF subunit SPT5, causes widespread RNA polymerase II to pause at gene promoters, thereby promoting cell cycle arrest. As a functional consequence of the loss of CDK9 activity, *MYC* expression is elevated, which in turn activates *EZH2*, a subunit of the PRC2 complex, resulting in methyltransferase activity and hence H3K27me3 reduction [30] (Fig. 3E).

### F. Fingerprint Global Chromatin Profiling Reveals Crosstalk Among “Regulators” in Breast Cancer Signaling Pathways

**Fig. 4.**
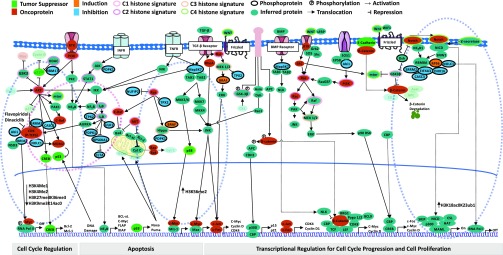
Fingerprint global chromatin profiling in breast cancer signaling. The crosstalk among histone signature pathways is depicted by linking inferred proteins/protein complexes generated from STRINGdb PPI for signaling pathways that may interact with the CDK pathway in cancer signaling landscape. As part of cell cycle regulation, inhibition of CDK by flavopiridol and dinaciclib is highlighted showing molecular cascades of interactions among BRD4, NSD3, SRRM2, NOLC1, MYC with the P-TEFb complex and its recruitment to promoter region to block transcriptional elongation of RNA Pol II (the first blue dashed oval). As a consequence, reduced levels of H3K27me3K36me3, H3K4me1, and H3K4me2 are observed. Examples of crosstalk includes: Ikkb inhibiting TPX2 which binds to AURKA to activate CDK targeting the intrinsic kinase activity directed towards RNA Pol II (pink dashed oval); reduction of Map3K7 brings JNK level down resulting in an increase of H3K56me2 level; hyperactive RAS acts as a signaling switch to convert JNK's role from pro- to anti-tumor signaling through the regulation of Hippo signaling activity by inhibiting PDPK1 protein (the second blue dashed oval). NOLC1 interacts with the EJC junction formed by SRRM2, CASC3, EIF4A3, and RBM8A proteins. It mediates Wnt/Notch signaling activity through the Notch intracellular domain (NICD) and monoubiquitylation of H3K23 (H3K18ac0K23ub1) by translocating to RNA Pol I (the third blue dashed oval).The color of molecules represents tumor suppressors (green), oncoproteins (red), inferred proteins (teal) and phosphoproteins (black ring). The color of protein molecule indicates whether the protein was induced (orange) or inhibited (blue). Each histone signature (C1, C2, C3, and C4) is highlighted using a distinct outer ring color. Phosphorylation, activation, and repression are indicated by Ⓟ, arrowheads (→), and cross-bars (⊣), respectively.

Finally, to highlight potential BRD4 mediated off-target effects of flavopiridol and dinaciclib, we constructed a detailed view of the crosstalk among the various regulators. We accomplished this by generating protein-protein interactions using STRINGdb to incorporate inferred proteins/protein complexes for other signaling pathways interacting with the CDK pathway. The detailed view of the breast cancer signaling landscape reveals various regulators associated with specific signaling pathways mediating cellular activities such as cell cycle regulation, apoptosis and transcriptional regulation for cell cycle progression and cell proliferation (Fig. 4, **Supplementary Table 6**). Collectively, these results indicate that BRD4 is an atypical kinase that could interact with a diverse group of kinases resulting in pleiotropic effects when treated with flavopiridol and dinaciclib.

## III. Discussion

Global chromatin fingerprints represent a new way to identify response of tumor cells to drug treatments. Further, the GCP also serve as endpoints of mechanisms responding to drugs and has the potential to provide insights into the detailed networks and their perturbations. Using iPhDNet, we were able to identify four distinct histone signatures and enriched phosphoproteins that contribute to specific histone codes. While our network shows multiple drugs and phosphoproteins regulating H3K27me3K36me3, flavopiridol, and dinaciclib demonstrated selective inhibitory effects on chromatin reader BRD4 modulating H3K27me3K36me3. In particular, our study implicates H3K27me3K36me3 as a potential biomarker that is targeted by flavopiridol and dinaciclib to induce cell cycle alterations by BRD4.

From the MCN analyses, we infer that BRD4 enrichment is maintained across 3, 6, 24-hour time points in flavopiridol, which lead us to conclude that the cell cycle arrest is induced via direct BRD4 mediation during G1/S phase as well as during late mitosis, G2/M transition. With dinaciclib, however, we observe enrichment of the EJC regulators involved in spliceosome related activities at 3 and 6-hour time points, which indicates cell cycle arrest most likely occurred due to nonsense-mediated mRNA decay (NMD) during G1/S phase, and indirect BRD4 mediation during G2/M phase (Fig. 4E). From these analyses, we can link cell cycle control to cell cycle arrest through the presence of an alternative splicing network. When treated with dinaciclib, we observe EJC members *SRRM2*, *CASC3* and *EIF4A3* interact with both upstream transcription factors UPF1, a known regulator of NMD, and ILF3. These transcription factors are known to modulate the Wnt/Notch signaling pathway through NMD and are highly active in pluripotent cells [31], suggesting possible influences in cellular state remodeling. We also observed enrichment of *AURKA* and *TPX2* regulators which are known to modulate cell program death via *BCL-x*, a *BCL2* family apoptosis regulator [32].

From the MCN analysis, we further observe the presence of a super-enhancer binding gene *OCT4* upstream of BRD4 suggesting the possible role of BRD4 in regulating pluripotency gene expression by exhibiting a “stemness” behavior. Previous studies have shown positive correlation between BRD4 and the level of H3K36me3 with *OCT4* [33]. Depletion of BRD4 has been shown to decrease the pluripotency of *OCT4* by changing the cellular fate through disruption of signaling pathways controlling differentiation [33]. Our analysis shows that, BRD4 interacts with the transcription factor SMARCA4, a key regulator of ESC self-renewal and pluripotency, known to regulate *NANOG* expression [33]. *NANOG* interacts directly with *OCT4*, and *SOX2* genes, which are pioneer transcription factors that maintain a pluripotent cell state [34]. This makes H3K36me3 a potential biomarker, regulated by the super enhancer-mediated BRD4 to study tumor transformation, tumorigenesis, and metastasis in breast cancer as well as in other cancers.

The global chromatin profiling fingerprints of the breast cancer landscape reveal crosstalk among various signaling pathways belonging to specific histone signatures, suggesting possible combinatorial targeted therapies to address off-target effects. In particular, these fingerprints show crosstalk between CDK and IkB signaling pathways involving interactions between P-TEFb and AURKA. Overexpression of AURKA is linked to many cancers. Our transcriptomic results show down-regulation of AURKA when treated with flavopiridol and dinaciclib, further suggesting the efficacy and ability of these drugs to minimize off-target effects in breast cancer.

## IV. Conclusion

We developed an integrative framework for the analysis of multi-omics data in deriving signatures of drug response in breast cancer. This framework, iPhDNet can be extended to include other data such as ATAC-seq and Hi-C. The methods applied reduce large multi-dimensional measurements into mechanistically insightful modules and provide fingerprints of cellular response. In our studies on drug treatments in breast cancer, we show the histone signatures that are both predictive of response and provide potential mechanism-driven therapeutic targets. In this study, response to flavopiridol and dinaciclib, breast cancer cells MCF7 through the activation of CDK inhibitor pathways and the chromatin reader BRD4 alter the histone marks H3K27me3K36me3. We show the altered regulation of the cell cycle. Overall, the integrative frameworks we developed reveal the mechanisms of action of specific drugs on chromatin remodeling machinery in breast cancer cells which lay the foundation for improved diagnostics and development of chromatin-based cancer therapy.

## V. MATERIALS AND METHODS

### A. Experimental Data Acquisition and Preprocessing

All experimental data was obtained from the LINCS and TCGA consortia. As described in the Supplementary Methods, data were preprocessed, normalized and missing data were imputed.

### B. iPhDNet

The components of iPhDNet include: NMF, PLSR, and series of statistical testing methods. All network reconstruction was done using correlation/connectivity methods and visualization was carried out using Cytoscape. A very detailed description of iPhDNet is presented in the Supplementary Methods Section.

## Supplementary Materials



The supplementary material includes descriptions of the data acquisition, preprocessing, transcriptomic analysis, experimental validation, and methods used to construct iPhDNet. It includes 3 supplemental figures and 6 tables.

## Supplementary Material


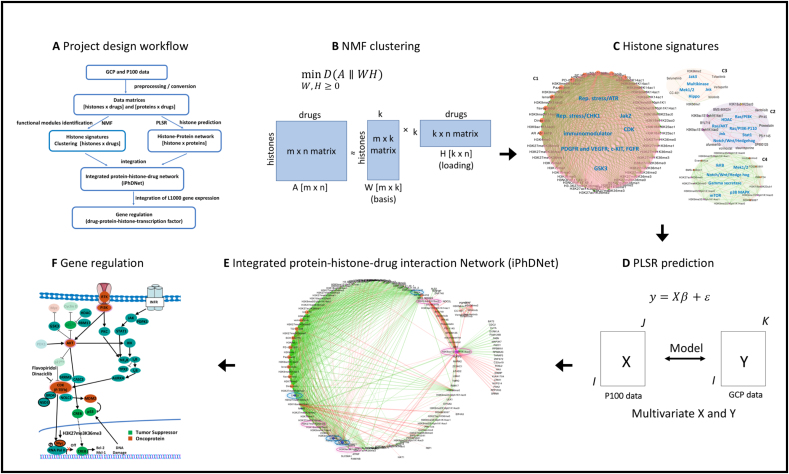
2.6 MBunix

Our study demonstrates the value of network-based global chromatin fingerprints to understand how unique alterations in histone modifications when treated with specific drugs, contribute to cellular reprogramming in breast cancer.

## References

[1] Hanahan D. and Weinberg R. A., “Hallmarks of cancer: The next generation,” Cell, vol. 144, no. 5, pp. 646–674, Mar. 2011. 21376230 10.1016/j.cell.2011.02.013

[2] Baylin S. B. and Jones P. A., “A decade of exploring the cancer epigenome––Biological and translational implications,” Nat. Rev. Cancer, vol. 11, no. 10, pp. 726–734, Sep. 2011. 21941284 10.1038/nrc3130 PMC3307543

[3] Sandoval J. and Esteller M., “Cancer epigenomics: Beyond genomics,” Curr. Opin. Genet. Dev., vol. 22, no. 1, pp. 50–55, Feb. 2012. 22402447 10.1016/j.gde.2012.02.008

[4] Leroy G. , “A quantitative atlas of histone modification signatures from human cancer cells,” Epigenetics Chromatin, vol. 6, no. 1, pp. 1–14, Jul. 2013. 23826629 10.1186/1756-8935-6-20 PMC3710262

[5] Strahl B. D. and Allis C. D., “The language of covalent histone modifications,” Nature, vol. 403, no. 6765, pp. 41–45, Jan. 2000. 10638745 10.1038/47412

[6] Foss F. M., Zinzani P. L., Vose J. M., Gascoyne R. D., Rosen S. T., and Tobinai K., “Peripheral T-cell lymphoma,” Blood, vol. 117, no. 25, pp. 6756–6767, Jun. 2011. 21493798 10.1182/blood-2010-05-231548

[7] Khan O. and La Thangue N. B., “HDAC inhibitors in cancer biology: Emerging mechanisms and clinical applications,” Immunol. Cell Biol., vol. 90, no. 1, pp. 85–94, Jan. 2012. 22124371 10.1038/icb.2011.100

[8] Abelin J. G. , “Reduced-representation phosphosignatures measured by quantitative targeted MS capture cellular states and enable large-scale comparison of drug-induced phenotypes,” Mol. Cell. Proteomics, vol. 15, no. 5, pp. 1622–1641, 5 2016. 26912667 10.1074/mcp.M116.058354 PMC4858944

[9] Subramanian A. , “A next generation connectivity map: L1000 platform and the first 1,000,000 profiles,” Cell, vol. 171, no. 6, pp. 1437–1452, Nov. 2017. 29195078 10.1016/j.cell.2017.10.049 PMC5990023

[10] Creech A. L. , “Building the Connectivity Map of epigenetics: Chromatin profiling by quantitative targeted mass spectrometry,” Methods, vol. 72, pp. 57–64, Jan. 2015. 25448295 10.1016/j.ymeth.2014.10.033 PMC4300274

[11] Lee D. D. and Seung H. S., “Learning the parts of objects by non-negative matrix factorization,” Nature, vol. 401, no. 6755, pp. 788–791, Oct. 1999. 10548103 10.1038/44565

[12] Boutsidis C. and Gallopoulos E., “SVD based initialization: A head start for nonnegative matrix factorization,” Pattern Recognit., vol. 41, no. 4, pp. 1350–1362, Apr. 2008.

[13] Krämer N. and Sugiyama M., “The degrees of freedom of partial least squares regression,” J. Amer. Stat. Assoc., vol. 106, no. 494, pp. 697–705, Jun. 2011.

[14] Lewis P. W. , “Inhibition of PRC2 activity by a gain-of-function H3 mutation found in pediatric glioblastoma,” Science, vol. 340, no. 6134, pp. 857–861, 5 2013. 23539183 10.1126/science.1232245 PMC3951439

[15] Zhu J. , “Gain-of-function p53 mutants co-opt chromatin pathways to drive cancer growth,” Nature, vol. 525, no. 7568, pp. 206–211, Sep. 2015. 26331536 10.1038/nature15251 PMC4568559

[16] Holm K. , “Global H3K27 trimethylation and EZH2 abundance in breast tumor subtypes,” Mol. Oncol., vol. 6, no. 5, pp. 494–506, Oct. 2012. 22766277 10.1016/j.molonc.2012.06.002 PMC5528390

[17] Yang X. , “CDKN1C (p57KIP2) is a direct target of EZH2 and suppressed by multiple epigenetic mechanisms in breast cancer cells,” PLoS One, vol. 4, no. 4, Apr. 2009, Art. no. e5011. 10.1371/journal.pone.0005011 PMC2659786 19340297

[18] Wang C. , “Expression of Abl interactor 1 and its prognostic significance in breast cancer: A tissue-array-based investigation,” Breast Cancer Res. Treat., vol. 129, no. 2, pp. 373–386, Sep. 2011. 21046228 10.1007/s10549-010-1241-0

[19] Dispenzieri A. , “Flavopiridol in patients with relapsed or refractory multiple myeloma: A phase 2 trial with clinical and pharmacodynamic end-points,” Haematologica, vol. 91, no. 3, pp. 390–393, Mar. 2006. 16503551

[20] Paruch K. , “Discovery of Dinaciclib (SCH 727965): A Potent and Selective Inhibitor of Cyclin-Dependent Kinases,” ACS Med. Chem. Lett., vol. 1, no. 5, pp. 204–208, Aug. 2010. 24900195 10.1021/ml100051d PMC4007794

[21] Ember S. W. J. , “Acetyl-lysine binding site of bromodomain-containing protein 4 (BRD4) interacts with diverse kinase inhibitors,” ACS Chem. Biol., vol. 9, no. 5, pp. 1160–1171, 5 2014. 24568369 10.1021/cb500072z PMC4032195

[22] Rahman S. , “The Brd4 extraterminal domain confers transcription activation independent of pTEFb by recruiting multiple proteins, including NSD3,” Mol. Cell. Biol., vol. 31, no. 13, pp. 2641–2652, Jul. 2011. 21555454 10.1128/MCB.01341-10 PMC3133372

[23] Wen H. , “ZMYND11 links histone H3.3K36me3 to transcription elongation and tumour suppression,” Nature, vol. 508, no. 7495, pp. 263–268, Apr. 2014. 24590075 10.1038/nature13045 PMC4142212

[24] Li F. , “The histone mark H3K36me3 regulates human DNA mismatch repair through its interaction with MutSα,” Cell, vol. 153, no. 3, pp. 590–600, Apr. 2013. 23622243 10.1016/j.cell.2013.03.025 PMC3641580

[25] Chen E. Y. , “Enrichr: Interactive and collaborative HTML5 gene list enrichment analysis tool,” BMC Bioinf., vol. 14, pp. 1–14, Apr. 2013. 10.1186/1471-2105-14-128 PMC3637064 23586463

[26] Sengupta S., Biarnes M. C., and Jordan V. C., “Cyclin dependent kinase-9 mediated transcriptional de-regulation of cMYC as a critical determinant of endocrine-therapy resistance in breast cancers,” Breast Cancer Res. Treat., vol. 143, no. 1, pp. 113–124, Jan. 2014. 24309997 10.1007/s10549-013-2789-2 PMC3908445

[27] Horiuchi D. , “MYC pathway activation in triple-negative breast cancer is synthetic lethal with CDK inhibition,” J. Exp. Med., vol. 209, no. 4, pp. 679–696, Apr. 2012. 22430491 10.1084/jem.20111512 PMC3328367

[28] Lu H. , “Correction: Compensatory induction of MYC expression by sustained CDK9 inhibition via a BRD4-dependent mechanism,” Elife, vol. 4, Jul. 2015, Art. no. e09993. 10.7554/eLife.09993 PMC4502752 26186095

[29] Curtis C. , “The genomic and transcriptomic architecture of 2,000 breast tumours reveals novel subgroups,” Nature, vol. 486, no. 7403, pp. 346–352, Apr. 2012. 22522925 10.1038/nature10983 PMC3440846

[30] Cha T.-L. , “Akt-mediated phosphorylation of EZH2 suppresses methylation of lysine 27 in histone H3,” Science, vol. 310, no. 5746, pp. 306–310, Oct. 2005. 16224021 10.1126/science.1118947

[31] Lou C.-H. , “Nonsense-mediated RNA decay influences human embryonic stem cell fate,” Stem Cell Rep., vol. 6, no. 6, pp. 844–857, Jun. 2016. 10.1016/j.stemcr.2016.05.008 PMC4912386 27304915

[32] Moore M. J., Wang Q., Kennedy C. J., and Silver P. A., “An alternative splicing network links cell-cycle control to apoptosis,” Cell, vol. 142, no. 4, pp. 625–636, Aug. 2010. 20705336 10.1016/j.cell.2010.07.019 PMC2924962

[33] Di Micco R. , “Control of embryonic stem cell identity by BRD4-dependent transcriptional elongation of super-enhancer-associated pluripotency genes,” Cell Rep., vol. 9, no. 1, pp. 234–247, Oct. 2014. 25263550 10.1016/j.celrep.2014.08.055 PMC4317728

[34] Liu A., Yu X., and Liu S., “Pluripotency transcription factors and cancer stem cells: Small genes make a big difference,” Chin. J. Cancer, vol. 32, no. 9, pp. 483–487, Sep. 2013. 23419197 10.5732/cjc.012.10282 PMC3845564

[35] Brunet J.-P., Tamayo P., Golub T. R., and Mesirov J. P., “Metagenes and molecular pattern discovery using matrix factorization,” Proc. Natl. Acad. Sci. U.S.A., vol. 101, no. 12, pp. 4164–4169, Mar. 2004. 15016911 10.1073/pnas.0308531101 PMC384712

[36] Sokal R. R. and Rohlf F. J., “The comparison of dendrograms by objective methods,” Taxon, vol. 11, no. 2, pp. 33–40, 1962.

[37] Gupta S., Maurya M. R., and Subramaniam S., “Identification of crosstalk between phosphoprotein signaling pathways in RAW 264.7 macrophage cells,” PLoS Comput. Biol., vol. 6, no. 1, Jan. 2010, Art. no. e1000654. 10.1371/journal.pcbi.1000654 PMC2813256 20126526

[38] Baldi P. and Long A. D., “A Bayesian framework for the analysis of microarray expression data: Regularized t -test and statistical inferences of gene changes,” Bioinformatics, vol. 17, no. 6, pp. 509–519, Jun. 2001. 11395427 10.1093/bioinformatics/17.6.509

[39] Kayala M. A. and Baldi P., “Cyber-T web server: Differential analysis of high-throughput data,” Nucleic Acids Res., vol. 40, no. Web Server issue, pp. W553–W559, Jul. 2012. 22600740 10.1093/nar/gks420 PMC3394347

