# Supplementary material for: Histone Signatures Predict Therapeutic Efficacy in Breast Cancer
Source: IEEE Open J Eng Med Biol. 2020 Jan 17;1:74–82. doi: 10.1109/OJEMB.2020.2967105 (PMC7207876; doi:10.1109/OJEMB.2020.2967105)
Supplement: Supplementary file 1 [file supp1-2967105.pdf]

Supplementary Materials

Histone Signatures Predict Therapeutic Efficacy in Breast Cancer

Shamim A. Mollah, Shankar Subramaniam, *Member, IEEE*

A. Histone, phosphoproteomic and transcriptomic data acquisition

The experimental data were generated by the NIH LINCS Proteomic Characterization Center for Signaling and Epigenetics (PCCSE) repository. Level 3 (log 2 normalized) targeted phosphoproteomics assay (P100) against 96 phosphopeptides data, and level 3 (log 2 normalized) global chromatin profiling assay (GCP) against 60 probes that monitor combinations of post-translational modification on histones data using various cancer cell lines including MCF7 (breast), YAPC (pancreas), A375 (melanoma), PC3 (prostate), A549 (lung) and NPC (Neural Progenitor) were downloaded. These assays were treated with 31 serine/threonine kinase inhibitors (drugs) at various concentrations, DMSO as a negative control and consisted of three biological replicates. Three time points (3, 6, 24 hour) were available for P100 data while a single time point (24 hour) was available for GCP data in MCF7 cells. Single time point (24 hour) was available for GCP data in YAPC, A375, PC3, A549, and NPC cell lines (**Supplementary Table 1**).

The experimental transcriptomic data was generated by the NIH LINCS Connectivity Map (CMap) using microarray platform. This assay, which is known as L1000, contained 978 landmark transcripts whose expressions were invariant across cell states. Level 3 (log 2 normalized) L1000 data for breast cancer cell line MCF7 were downloaded.

Patient-level data were downloaded from The Cancer Genome Atlas (TCGA) where breast tissue samples were obtained from 113 normal patients and breast cancer tissue samples were obtained from 303 ER+/HR+/HER2- cancer patients from molecular taxonomy of breast cancer international consortium (METABRIC) study [29] (**Supplementary Table 4**).

B. Data pre-processing

Replicates were used to impute missing data by taking their weighted average values during the pre-processing step. Differential histone modifications and phosphorylation changes were computed by taking fold changes of each perturbed phosphopeptide and histone code with respect to DMSO. These resulted in two data matrices, i) phosphoprotein profiles consisting of [96 peptides x 31 drugs], and ii) global chromatin profiles consisting of [60 histone modifications x 31 drugs]. Prior to modeling, P100 data were normalized with respect to the mean and standard deviation of the respective variables.

C. Experimental Validation

L1000 genes expressions were used to validate differential gene expressions of the 31 functionally significant genes (cell cycle genes, CDK inhibitor gene *CDKN2A*, transcription factor *MYC* and genes representing the enriched phosphoproteins) to capture in vitro gene activity levels in (MCF7) cell line.

D. Quantification and Statistical Analysis

Histone Signature Identification

An unsupervised clustering technique, non-negative matrix factorization (NMF), was used to stratify histone signatures. R Statistics package was used for the calculation and Cytoscape was used to generate network graphs. Similar to vector quantization methods such as principal components analysis (PCA) and singular value decomposition (SVD), the objective of NMF is to explain the observed data using a compact number of latent features, i.e., basis components, which when combined with loading/mixture components approximate the original data as accurately as possible. In our NMF formulation both the matrix representing the basis components (histone signatures) as well as the matrix of mixture coefficients (drug prototypes) are constrained to have non-negative values, and unlike PCA and SVD, no independence or orthogonally constraints are imposed on the basis components leading to a simple and intuitive interpretation of the factors that allow the basis components to overlap. This unique feature is particularly interesting in histone modules, where overlapping basis components identify combinatorial histone codes resulting from multiple signaling pathways and indicate a specific signature. Because NMF assumes an additive model, anti-log transformed values were used in our analysis.

Mathematically, NMF consists of finding an approximation

$$A \approx WH, \quad W, H \geq 0 \qquad (1)$$

where W, H are n × k and k × m non-negative matrices respectively where n are rows – samples and m are columns – the measured features in A. Since the objective is to reduce the dimension of the original data A, the factorization rank k is often chosen such that k << (n, m). **W** contains basis vectors and **H** contains encoding vectors that estimate the extent to which each basis vector is used to reconstruct each input vector. We used a version of NMF to minimize the divergence function (KL divergence) given by Brunet et al. [35]. The function is related to the Poisson likelihood of generating A from W and H, more specifically, based on randomly initialized matrices **W** and **H**, NMF finds the solution of

$$\min D(\mathbf{A} \| \mathbf{WH}) = \sum_{i=1}^{n} \sum_{j=1}^{m} \left(A_{ij} \log \frac{A_{ij}}{(WH)_{ij}} - A_{ij} + (WH)_{ij}\right) \qquad (2)$$

where, D is a loss function, via an iterative process [11]. At each step, W and H are updated by using the following coupled divergence equations:

$$H_{a\mu} \leftarrow H_{a\mu} \frac{\sum_i W_{ia} A_{i\mu}/(WH)_{i\mu}}{\sum_k W_{ka}} \quad (3)$$

$$W_{ia} \leftarrow HW_{ia} \frac{\sum_\mu WH_{a\mu} A_{i\mu}/(WH)_{i\mu}}{\sum_v H_{av}} \quad (4)$$

where $\mathbf{A}_{i,j} = [\mathbf{A}]_{i,j}$ indicates (i,j)-th element of the matrix **A**.
Because (1) is non-convex optimization with respects to W and H, there is no guarantee of obtaining a local minimum. Moreover, the above iterative update rules are notorious for slow convergence (i.e., require more iterations) and have a complexity of $O(mnk^2N_iN_o)$ where N_i is the number of inner iterations to solve the non-negative linear model and N_o is the number of outer iterations to alternate W and H steps. As a result, the initialization of the pair of factors (W, H) is considered an important component in the design of successful NMF methods [11]. We used a robust initialization strategy using the seeding algorithm, that is based on a non-negative double singular value decomposition (nndSVD) [12]. The whole process then becomes deterministic and needs to run once and the complexity is reduced to $O(mnk^2N_i + N_o)$. Our NMF framework works as follows:

1. Initialize W, H ∈ $R_{m\times k}$, $R_{k\times n}$ respectively with non-negative elements using nndSVD.

2. Repeat until a convergence criterion is satisfied:

$$H_{a\mu} \leftarrow H_{a\mu} \frac{\sum_i W_{ia} A_{i\mu}/(WH)_{i\mu}}{\sum_k W_{ka}}$$

$H \geq 0$
where W is fixed, and

$$W_{ia} \leftarrow HW_{ia} \frac{\sum_\mu WH_{a\mu} A_{i\mu}/(WH)_{i\mu}}{\sum_v H_{av}}$$

$W \geq 0$
where H is fixed

3. The columns of W are normalized and the rows of H are scaled accordingly.

E. Cluster Validation

To identify the optimal rank k, we used the cophenetic correlation coefficient [36] to determine the most robust clustering as:

$$c = \frac{\sum_{i<j}(x(i,j) - \bar{x})(t(i,j) - \bar{t})}{\sqrt{[\sum_{i<j}(x(i,j) - \bar{x})^2][\sum_{i<j}(t(i,j) - \bar{t})^2]}}. \quad (5)$$

It measures how reliably the same histone codes are assigned to the same cluster across many iterations of the clustering algorithm with random initializations. The cophenetic correlation coefficient lies between 0 and 1 and reflects the probability that samples i and j cluster together. Higher values indicate more stable cluster assignments. We selected optimal k= 4 (**Supplementary Fig S1A, S1B**) based on the largest observed cophenetic coefficient and where the magnitude of the cophenetic correlation begins to decrease by varying values of k from 2 to 10 (**Supplementary Fig S1C**). We used the NMF package in R to implement and compute these calculations.
In eq 5, $x(i, j) = |x_i - x_j|$, is the ordinary Euclidean distance between the *i*th and *j*th observations. $t(i, j)$ = the dendrogrammatic distance between the model points t_i and t_j (height of the node at which these two points are first joined), *x* bar is the average of the $x(i, j)$, and *t* bar is the average of the $t(i, j)$. After factorizing **A** into the basis matrix **W** and the *encoding* matrix **H**, we used the basis matrix **W** for histone stratification. Specifically, we grouped histone codes into *four groups (k=4). We assigned histone code x_i to cluster k* which has the highest value based on the basis vector, as:*

$$k^* = \arg\max_k W_{i,k} \quad (6)$$

Similarly, we assigned targeted pathways for each drug d_j to cluster k* which has the highest value based on the encoding vector, as:

$$k^* = \arg\max_k H_{j,k} \quad (7)$$

F. Histone Prediction Model

Histone-peptide interaction network was generated using partial least square regression (PLSR) method based on Kraemer et al. formulation [13]. PLSR is a multivariate regression method for constructing predictive model when the number of factors/predictor variables (in our case phosphopeptides) exceeds the number of responses / dependent variables (histone marks), and collinearity exists (phosphopeptides are correlated with one another). A past study [37] had shown the effectiveness of partial least square (PLS) application in understanding crosstalk between phosphoprotein signaling in macrophage cells, thus, prompting us to consider a PLS-based regression model. The general idea behind PLSR is to try to extract latent factors, accounting for as much of the observed variation as possible while modeling the responses well. For each sample n, the value y_{nj} is defined as:

$$y_{nj} = \sum_{i=0}^{k} b_i x_{ni} + \varepsilon_{nj} \quad (8)$$

Where y_{nj} is a response, b_i is the coefficient, x_{ni} is an explanatory variable and ε_{nj} is an error term. This model is similar to linear regression; however, the way these β_i are found is different. To see this, a matrix format of equation (7) can be expressed as ***Y=XB+E*** where ***Y*** is an *n* cases by *m* variables response matrix (in our case it is drugs x histone data), ***X*** is an *n* cases by *p* variables predictor matrix (in our case it is drugs x phosphopeptides data), ***B*** is a *p* by *m* regression coefficient matrix, and ***E*** is a noise term for the model which has the same dimensions as ***Y***. For our ***X*** predictor matrix, we first normalized all the phosphosignal values to their corresponding z-scores and centered ***Y*** response matrix (histone values). Intuitively, partial least squares regression produces a *p* by *c* weight matrix ***W*** for ***X*** such that ***T***=***XW***, i.e., the columns of ***W*** are weight vectors for the ***X*** columns producing the corresponding *n* by *c* factor score matrix ***T***. These weights are computed so that each of them maximizes the covariance between responses and the corresponding factor scores. Ordinary least squares procedures for the regression of ***Y*** on ***T*** are then performed to produce ***Q***, the loadings for ***Y*** (or weights for ***Y***) such that ***Y***=***TQ***+***E***. Once ***Q*** is

computed where $B=WQ$, we have $Y=XB+E$, and the prediction model is complete. To provide a complete description of PLSR, we also need a p by c factor loading matrix P which gives a factor model $X=TP+F$, where F is the unexplained part of the X scores.

On the training data, we calculated the optimal model parameter using 10-fold cross-validation. We assessed the predictive performance by computing the residual sum of square (RSS) error of prediction on the test set (**Supplementary Fig S2**).

We identified the optimal number of components (principal component, PC) that could be used to predict the model accurately using residual square sum (RSS) value < 0.05. Once the coefficients (β_i) are generated, we retained only the significant peptides (p_value < 0.0001) using a t-test where the degree of freedom DOF was computed as:

DOF = min (column of X, row of X) – PC – 1.

G. Integrated Phosphoprotein-Histone-Drug Network (iPhDNet)

Using the coefficients from the histone signatures (c1, c2, c3, and c4) and the drug prototypes using NMF and model coefficients of phosphoproteins towards histone model prediction using PLSR, an integrated 3D network file is constructed connecting drugs to phosphoproteins and phosphoproteins to histones (iPhDNet). iPhDNet is visualized using Cytoscape highlighting hub nodes (most connected histones) linking histone to phosphoproteins to drugs. Influences of each drug or phosphoprotein towards a histone code then can be visualized by the properties of edges connecting them. For example, the thickness of the edges signifies the amount of contributions by each phosphoprotein or drug, colors of edges signify how they are correlated (i.e., positive or negative).

H. Mechanistic Causal Network (MCN) Reconstruction

A time-varying mechanistic causal network was constructed by back propagating iPhDNet, previously generated for 24 hour from P100 data. We first used a one-way ANOVA with a p-value of 1.0e-4 to populate enriched (statistically significant) phosphoproteins at 6 and 3-hour time points. We then inferred protein-protein interactions for the phosphoproteins enriched in 24 hour by mapping them to the STRING database. An interaction score of 0.8 and above, experimentally validated PPIs, and gene fusions criteria were used to obtain these inferred proteins. Our final MCN was constructed by back propagating our mapping of the inferred proteins from 24 hour to enriched phosphoproteins in 6 and to 3 hour. Additional protein-coding genes were generated and added to the final MCN using the EnrichR tool (http://amp.pharm.mssm.edu/Enrichr/enrich). We then validated our MCN by matching them against significant differentially expressed (DE) genes in L1000. Cytoscape was used to view the final reconstructed MCN.

I. Transcriptomic Analyses - Differential Expression of L1000 and TCGA Data

Differential expression analyses for 978 landmark genes from L1000 assay treated with flavopiridol and dinaciclib were performed using the unpaired t-test implemented in CyberT. Cyber-T is based on a regularized Bayesian framework that addresses technology biases and low replication levels in high throughput data [38]. These analyses were performed on 3, 6 and 24-hour datasets. Multiple corrections were applied to p-values using Benjamini Hochberg. Similarly, differential expression analyses of TCGA matched normal vs cancer patients were performed using unpaired t-tests. Cyber-T web server [39] was used to generate these analyses (**Supplementary Table 4**).

J. Cluster Similarity Evaluation

We used the Rand Index (RI) to evaluate the similarity of cluster assignments between every paired treatments in breast cancer and other cell lines. RI computes the percentage of pairs of objects for which both classification methods, the computed and the ideal one, agree. It is computed using False Positives (FP), False Negatives (FN), True Positives (TP) and True Negatives (TN) as follows:

$$RI = \frac{(TP+TN)}{(TP+TN+FP+FN)} \quad (9)$$

The RI value ranges from 0 (completely dissimilar group assignment) to 1 (exactly same group assignment).

K. Software Availability

Genomic, transcriptomic, epigenetic, and proteomic data files are available from the public online portal (https://panoramaweb.org/project/LINCS/GCP/begin.view?). Source codes are implemented in R 3.3.1 and are freely available for download at (https://github.com/smollah/iPhDNet).

L. Supplementary Figures

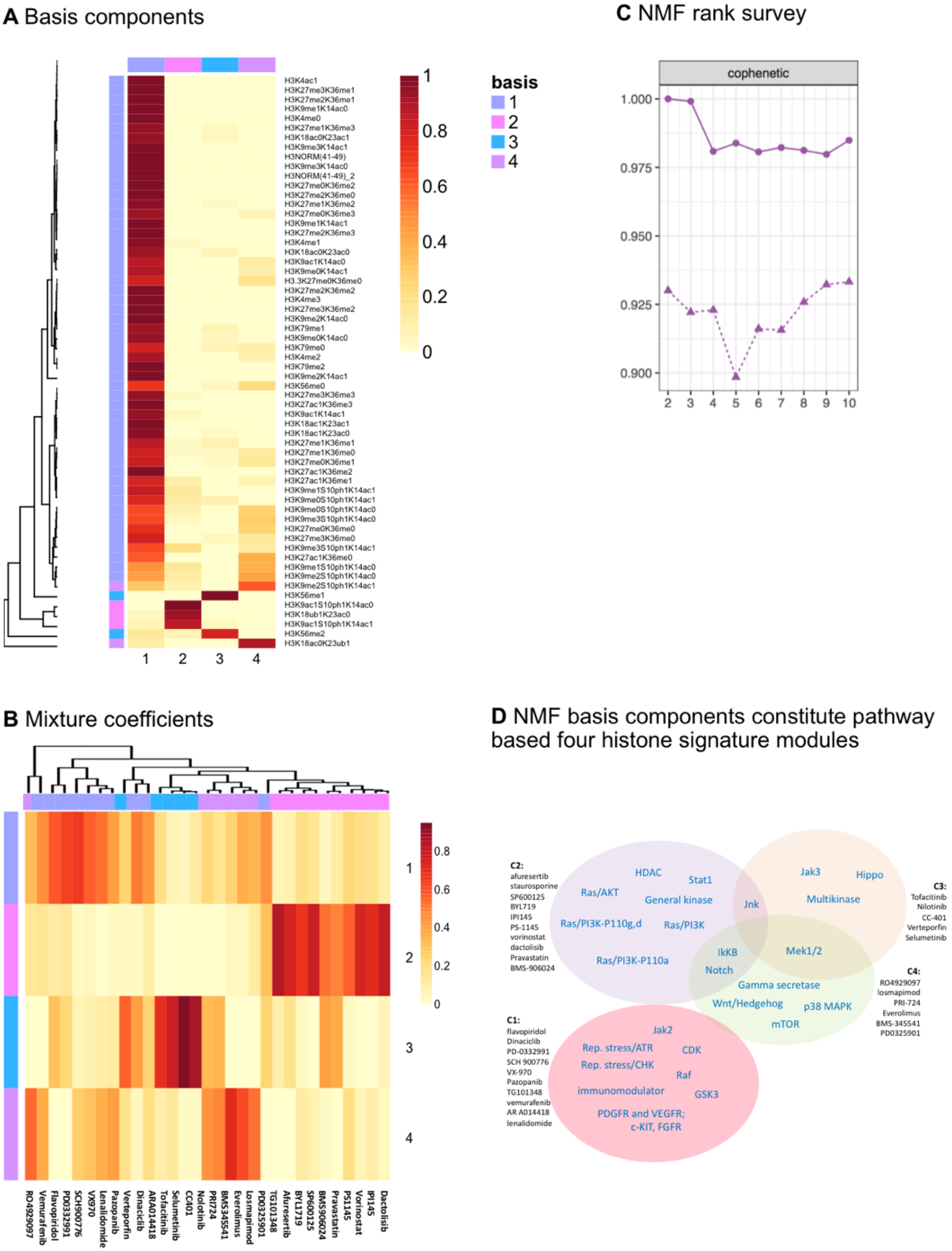

Fig S1. Estimation of the factorization rank of NMF and its cluster components. (**A**) Heatmap of the basis components (histones and their cluster memberships). Showing likelihood of each histone code belonging to a specific signature module. (**B**) Showing membership contributions of each drug toward 4 signature modules (k=4). (**C**) Cophenetic score is computed from 100 runs for each value of rank k by varying k= 2, 3...10 on 24-hour GCP data. Rank k represents the number of clusters or basis components. The solid line represents the original data and the dotted line represents random data. (**D**) Showing these 4 basis components corresponds to 4 pathway-based functional modules (c1, c2, c3 and c4). These functional modules constitute histone signatures.

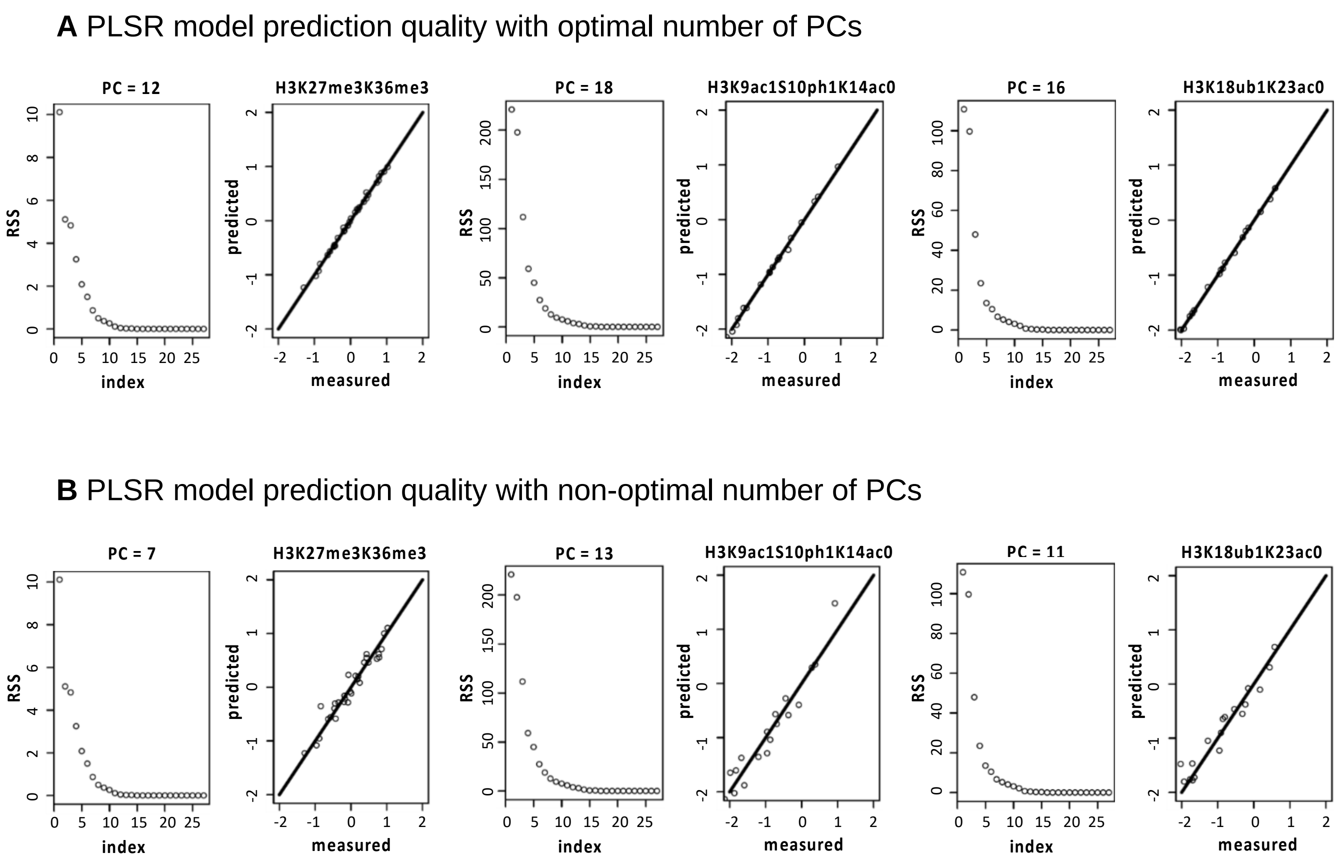

Fig S2. Performance of PLRS model, related to Figure1. Showing three examples of histone codes (H3K27me3K36me3, H3K9ac1S10ph1K14ac0 and H3K18ub1K23ac0). (**A**) Showing model performance using optimal number of components. The optimal number of components (principal component, PC) is used to predict the model accurately using residual square sum (RSS) value < 0.05. Once the coefficients (β_i) are generated, only the significant phosphoproteins (p_value < 0.0001) are retained using t-test. (**B**) Depicting model performance using sub optimal number of components for e.g., using 5 components fewer than the optimal component number.

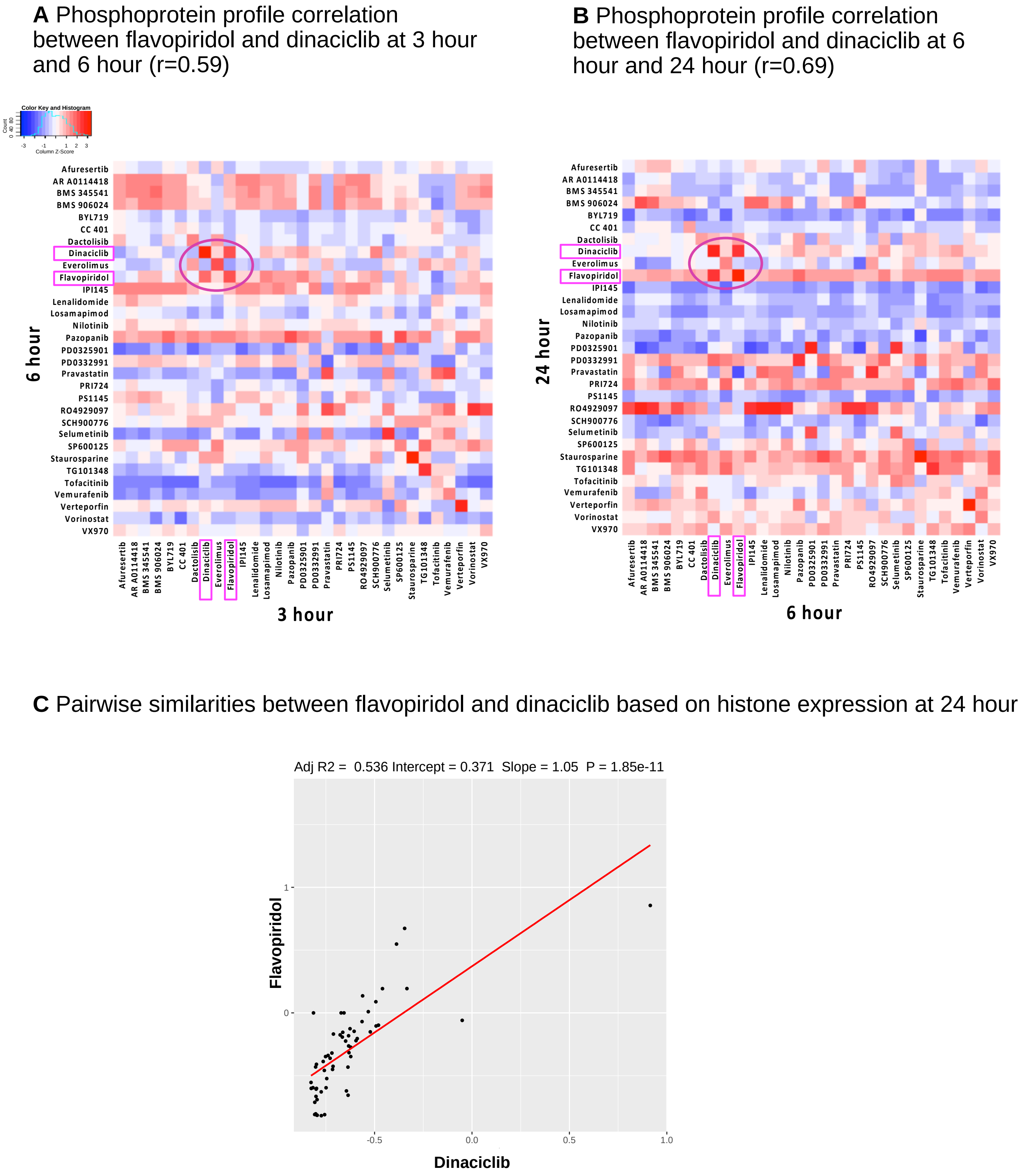

Fig S3. Phosphoprotein and global chromatin correlation profiles between drug pairs. (**A**) Pearson correlation between paired drugs at 3 and 6 hours. Showing a strong positive correlation (r=0.59) between flavopiridol and dinaciclib (circled) at 3 and 6 hours. (**B**) Strong positive correlation (r=0.69) is sustained between flavopiridol and dinaciclib (circled) at 6 and 24 hours. (**C**) Pairwise correlation between flavopiridol and dinaciclib based on 24 hour GCP data. Showing positive correlations between flavopiridol and dinaciclib (positive slope), using a linear regression line on 24 hour normalized global chromatin data (p-value = 1.85e-11, adjusted r-squared =0.536).

Supplementary Table 1. Library of Integrated Network-Based Cellular Signatures (LINCS) proteomics dataset used in our study

Cell line	GCP data	GCP time point	P100 data	P100 time point
MCF7 (Breast cancer)	LINCS_GCP_Plate29_annotated_minimized_2016-01-08_09-27-26_unprocessed.gct	24 hour	LINCS_P100_PRM_Plate29_03H_annotated_minimized_2016-01-28_11-00-43.gct<br>LINCS_P100_PRM_Plate29_06H_annotated_minimized_2016-01-28_17-11-17.gct<br>LINCS_P100_PRM_Plate29_24H_annotated_minimized_2016-01-28_17-11-22.gct	3, 6, 24 hour
YAPC (Pancreas cancer)	LINCS_P100_PRM_Plate32_annotated_minimized_2016-07-22_11-29-42.gct	24 hour		
A375 (Melanoma)	LINCS_GCP_Plate28_annotated_minimized_2016-04-14_14-24-24.gct	24 hour		
PC3 (Prostate cancer)	LINCS_GCP_Plate34_annotated_minimized_2016-07-07_14-16-01.gct	24 hour		
A549 (Lung cancer)	LINCS_GCP_Plate33_annotated_minimized_2016-06-03_14-58-02.gct	24 hour		
NPC (Neural Progenitor Cells)	LINCS_GCP_Plate27_annotated_minimized_2016-04-14_14-24-09.gct	24 hour		

Supplementary Table 2. Characteristics of the NMF based four histone signatures in MCF7

Histone signature	# of Histone codes	Histone codes	# of Drugs	Drugs	Canonical pathways
c1	46	H3K4me0, H3K4me1, H3K4me2, H3K4me3,H3K4ac1, H3K9me0K14ac0, H3K9me1K14ac0, H3K9me2K14ac0, H3K9me3K14ac0, H3K9ac1K14ac0, H3K9me0K14ac1, H3K9me1K14ac1, H3K9me2K14ac1, H3K9me3K14ac1, H3K9ac1K14ac1, H3K9me0S10ph1K14ac1, H3K9me1S10ph1K14ac1, H3K18ac0K23ac0, H3K18ac1K23ac0, H3K18ac0K23ac1, H3K18ac1K23ac1, H3K27me1K36me0, H3K27me1K36me1, H3K27me1K36me2, H3K27me1K36me3, H3K27me2K36me0, H3K27me2K36me1, H3K27me2K36me2, H3K27me2K36me3, H3K27me3K36me0, H3K27me3K36me1, H3K27me3K36me2, H3K27me3K36me3, H3K27ac1K36me1, H3K27ac1K36me2, H3K27ac1K36me3, H3.3K27me0K36me0, H3NORM.41.49_1, H3NORM.41.49_2, H3K56me0, H3K79me0, H3K79me1, H3K79me2, H3K27me0K36me1, H3K27me0K36me2, H3K27me0K36me3	10	AR-A014418, Dinaciclib, Flavopiridol, Lenalidomide, Pazopanib, PD-0332991, SCH900776, TG101348, Vemurafenib, VX970	GSK3 inhibitor, CDK/1,2,4,5,6,9 inhibitor, immunomodulator, PDGFR and VEGFR; Also c-KIT, FGFR, inhibitor, Rep. stress/CHK1 inhibitor, Jak2 inhibitor, Raf inhibitor, Rep. stress/ATR inhibitor
c2	3	H3K9ac1S10ph1K14ac0, H3K9ac1S10ph1K14ac1, H3K18ub1K23ac0	10	afuresertib, BMS906024, BYL719, dactolisib, IPI145, Pravastatin, PS-1145, SP600125, staurosporine, vorinostat	Ras/AKT inhibitor, Notch/other inhibitor, Ras/PI3K-P110a inhibitor, Ras/PI3K inhibitor, Ras/PI3K-P110g,d inhibitor, Stat1 inhibitor, IkKB inhibitor, Jnk inhibitor, Kinase inhibitor; general, HDAC inhibitor; general
c3	2	H3K56me1, H3K56me2	5	CC-401, Nilotinib, Selumetinib, Tofacitinib, Verteporfin	Jnk inhibitor, Multikinase inhibitor, Mek1/2 inhibitor, Jak3 inhibitor, Hippo inhibitor
c4	9	H3K9me0S10ph1K14ac0, H3K9me1S10ph1K14ac0, H3K9me2S10ph1K14ac0, H3K9me3S10ph1K14ac0, H3K9me2S10ph1K14ac1, H3K9me3S10ph1K14ac1, H3K18ac0K23ub1, H3K27me0K36me0, H3K27ac1K36me0	6	BMS-345541, Everolimus, losmapimod, PD0325901, PRI-724, RO4929097	IkKB inhibitor, mTOR inhibitor, p38 MAPK inhibitor, Mek1/2 inhibitor, Notch/Wnt/Hedgehog inhibitor, Notch/gamma secretase inhibitor

Supplementary Table 3. Characteristics of the enriched phosphoproteins

Phosphoprotein	Description	Function
BRD4	Bromodomain Containing 4	An epigenetic "reader" and belongs to BET family protein that maintains epigenetic memory and regulates cell cycle progression; BRD4 has been shown to have an intrinsic binding specificity for transcription factors such as c-MYC and p53 which are known to promote cancer (Delmore et al. 2011), making it a promising drug target.
ATAD2	ATPase Family, AAA Domain Containing 2	A bromodomain protein. It is a novel cofactor for MYC, overexpressed and amplified in aggressive tumors. It has been shown that downregulation of ATAD2 via siRNA results in increased apoptotic activity, suggesting a role for inhibitors of ATAD2 in cancer cell death and tumor regression (Caron et al. 2010).
NOLC1	Nucleolar and Coiled-Body Phosphoprotein 1	A nucleolar protein that regulates RNA polymerase I by connecting RNA polymerase I to ribosomal processing and remodeling enzymes, resulting in translational remodeling. It has a high binding affinity to c-MYC and Max transcription factors which play an important role in cancer. Although NOLC1 has not been studied extensively, a previous study found NOLC1 to have transcription factor-like activity in nasopharyngeal cancer progression suggesting its possible role in other cancers (Hwang et al. 2009).
SRRM2	Serine/Arginine Repetitive Matrix 2	Known to be involved in pre-mRNA splicing and has binding specificity for p53. SRRM2 has been detected as a 5'-3' Exoribonuclease 2 (Xrn2)-interacting protein that is involved in premature termination of RNA polymerase II transcription (Sansó et al. 2016; Brannan et al. 2012) thus affecting cell cycle progression.
CASC3	Cancer Susceptibility Candidate 3	Also known as MLN51 is a component of the exon junction complex (EJC) whose expression as been shown to be elevated in some breast cancer cell lines (Tomasetto et al. 1995).<br>The EJC is known to be involved in a surveillance mechanism that degrades mRNAs with premature translation termination codons through a nonsense-mediated mRNA decay (NMD) function, thereby, promoting cell cycle arrest.

Supplementary Table 4. Transcriptomic datasets used in our study.

L1000 data	Treatment	Concentration	L1000 time point	P100 data	Data Level
MCF7 (Breast cancer)	Flavopiridol	0.37 uM	3, 24 hour	Slicr_data_3_mcf7_alvocidib_dmso.zip<br>Slicr_data_24_mcf7_alvocidib_dmso.zip	3
		0.4 uM	6 hour	6hr_L1000_LEVEL4_n1667x978.txt(Jaffe et al)	4
	Dinaciclib	0.37 uM	24 hour	Slicr_data_24_mcf7_dinaciclib_dmso.zip	3
		0.4 uM	6 hour	6hr_L1000_LEVEL4_n1667x978.txt (Jaffe et al)	4
**Sample data**	**Patient derived data**		**Data Repository**		
Normal breast tissue	Matched normals, controls=113 (TCGA)		http://www.cbioportal.org/		
Breast cancer tissue	PAM 50 subtype: ER+, PR+, Her2-<br>METABRIC cohort, cases=303		Curtis C, et al., Nature (2012); Pereira B, et al., Nature communications (2016)<br>http://www.cbioportal.org/		

Supplementary Table 5. Summary results from MCN and Enrichment analysis

Regulators	Drug	Observations / characteristics
BRD4, TMPO, FAM76B, RBM17	Flavopiridol	Remained enriched across 3, 6, and 24 hour time points
TMPO, FAM76B, TPX2	Dinaciclib	Remained enriched across 3, 6, and 24 hour time points
BRD4, NSD3, H3K36 methylation	Flavopiridol, Dinaciclib	Binding of BRD4/NSD3 which is consistent with a previous study where they found that reduced H3K36 methylation was a result of depletion of BRD4 or NSD3 (Rahman et al., 2011).
NSD3, BRD4, H3K36me3	Flavopiridol, Dinaciclib	Nuclear receptor SET domain-containing 3 (NSD3), also known as WHSCL1, is a methyltransferase that binds to BRD4 complexes at the promoter region to regulate levels of H3K36me3, affecting DNA repair, transcription initiation and elongation / termination process (Wen et al., 2014; Li et al., 2013).
MYC, POU5F1, ESR2, UPF1 SMARCA4, BRCA1	Flavopiridol, Dinaciclib	The enrichment analysis identified these as commonly enriched upstream/core regulators of phosphoproteins for these drugs.
MYC, POU5F1, ESR2, UPF1 SMARCA4, BRCA1	Flavopiridol, Dinaciclib	These core regulatory factors have been shown to interact with super-enhancer genes which are master transcription factors that control cell identity by exhibiting higher sensitivity to transcription activities (Whyte et al., 2013).
POU5F1, H3K36me3	Flavopiridol, Dinaciclib	POU5F1 (OCT4) is a pioneer transcription factor whose expression has been shown to have association with a high level of H3K36me3 active mark (Musselman et al., 2012).
BRD4	Flavopiridol, Dinaciclib	BRD4 is reduced. Reduction of BRD4 is linked to reduced transcription of genes at super-enhancers (Loven et al. 2013). This further implicates the efficacy of flavopiridol and dinaciclib targeting BRD4 in breast cancer
E2F4, UPF1, ILF3, SMARCA4	Flavopiridol, Dinaciclib	Enrichment analysis showed interactions between spliceosome mediated activities through these core regulators and the components of exon junction complex (EJC), comprised of enriched genes namely, *NOLC1, SRRM2, CASC3, EIF4A3* and *RBM8A* (Le et al., 2016). EJC has been shown to have association with Wnt/Notch signaling activity in the cancer signaling pathway (Liu, et al., 2016)suggesting a crosstalk among pathways with possible off-target effects.
TPX2, AURKA, TP53, ATAD2	Flavopiridol, Dinaciclib	The enrichment analysis showed interactions among the mitotic regulators (*TPX2, AURKA*) with TP53 activity and *ATAD2* that formed a cluster, regulating cell cycle through alternative splicing.
TPX2, AURKA, EJC, BRD4	Flavopiridol, Dinaciclib	TPX2, AURKA and EJC complex are potential substrates of positive transcription elongation factor's (P-TEFb), which bind indirectly with BRD4.
ESR1, ESR2, SRRM2, NOLC1, MYC	Flavopiridol, Dinaciclib	Observed enrichment of estrogen receptors ESR1 and ESR2 as upstream regulators for SRRM2 and NOLC1 supporting possible MYC mediated endocrine activities.
MYC, SRRM2, NOLC1, BRD4	Flavopiridol, Dinaciclib	A recent study showed high MYC transcription mediated by CDK9 as a critical determinant of endocrine-therapy resistance breast cancers (Sengupta et.al, 2014). Therefore, it is reasonable to postulate that inhibition of SRRM2 and NOLC1 which interact with BET proteins may prove to be efficacious for endocrine therapy refractory breast cancers in a clinical setting.

Supplementary Table 6. Results of Crosstalk among regulators in breast cancer signaling pathway

Regulator	Affected pathway
BRD4, NSD3, SRRM2, NOLC1, MYC, P-TEFb complex	Inhibition of CDK by flavopiridol and dinaciclib shows molecular cascades of interactions among BRD4, NSD3, SRRM2, NOLC1, MYC with the P-TEFb complex and its recruitment to the proximal promoter region of MYC to block transcriptional elongation of RNA Pol II.
AURKA, TPX2	Presence of crosstalk among CDK, IkK, AKT, PI3K, and Map3K7 pathways when P-TEFb binds to AURKA, TPX2, and other proteins.
P-TEFb, AURKA	IkB, an enzyme complex that is part of the NF-κB signaling pathway, interacts with P-TEFb via AURKA to activate the CDK pathway. P-TEFb targets the intrinsic kinase activity directed towards RNA Pol II essential for transcriptional initiation, elongation, and inhibition.
BRD4, P-TEFb, RELA	BRD4 has been implicated in activating NF-κB pathway by recruiting P-TEFb to acetylated RELA. (Huang et al. 2009)
BRD4, H3K56me2	As a consequence of the CDK and BRD4 inhibition, we observed a reduction of Map3K7 phosphorylation, which inhibits JNK expression resulting in an increase of H3K56me2 level.
PDPK1	Hyperactive RAS then acts as a signaling switch that converts JNK's role from pro- to anti-tumor signaling through the regulation of Hippo signaling activity<br>by inhibiting the PDPK1 phosphoprotein.
BET proteins	A recent study has shown that the combined effect of PI3K and BET inhibition in a wide range of cancer cell lines resulted in apoptosis, tumor regression, and clamped inhibition of PI3K signaling. (Stratikopoulos et al., 2015)
EJC, P-TEFb, BRD4, RBM8A, ZC3H18	While EJC regulators indirectly bind to P-TEFb recruited by BRD4 via RBM8A and ZC3H18, they have a secondary binding effect with Wnt/Notch signaling pathway components.
SRRM2, CASC3, EIF4A3, RBM8A, NOLC1, H3K18ac0K23ub1	We observed NOLC1 interacted with the EJC regulators: SRRM2, CASC3, EIF4A3 and RBM8A proteins; Thus, we postulate that NOLC1 mediates Wnt/Notch signaling activity through Notch intracellular domain (NICD) and monoubiquitylation of H3K23 (H3K18ac0K23ub1) by translocating to RNA Pol I.
